# Demographic characteristics and clinical features of patients presenting with different forms of cutaneous leishmaniasis, in Lay Gayint, Northern Ethiopia

**DOI:** 10.1371/journal.pntd.0012409

**Published:** 2024-08-15

**Authors:** Endalew Yizengaw, Bizuayehu Gashaw, Mulat Yimer, Yegnasew Takele, Endalkachew Nibret, Gizachew Yismaw, Edward Cruz Cervera, Kefale Ejigu, Dessalegn Tamiru, Abaineh Munshea, Ingrid Müller, Richard Weller, James A. Cotton, Lloyd A. C. Chapman, Pascale Kropf

**Affiliations:** 1 Department of Medical Laboratory Science, College of Medicine and Health Science, Bahir Dar University, Bahir Dar, Ethiopia; 2 Institute of Biotechnology, Bahir Dar University, Bahir Dar, Ethiopia; 3 Amhara Public Health Institute, Bahir Dar, Ethiopia; 4 Department of Biology, College of Science, Bahir Dar University, Bahir Dar, Ethiopia; 5 Department of Infectious Disease, Imperial College London, London, United Kingdom; 6 Nefas Mewcha Hospital, Lay Gayint, Ethiopia; 7 Department of Dermatology, University of Edinburgh, Edinburgh, United Kingdom; 8 School of Biodiversity, One Health and Veterinary Medicine, College of Medical, Veterinary and Life Sciences, University of Glasgow, Glasgow, United Kingdom; 9 Department of Mathematics and Statistics, Lancaster University, Lancaster, United Kingdom; Instituto Goncalo Moniz-FIOCRUZ, BRAZIL

## Abstract

Cutaneous leishmaniasis (CL) is a neglected tropical disease caused by *Leishmania* parasites, that can cause long-term chronic disabilities. The clinical presentation of CL varies in both type and severity. CL presents as three main clinical forms: localised lesions (localised cutaneous leishmaniasis, LCL); mucocutaneous leishmaniasis (MCL) that affects the mucosa of the nose or the mouth; or as disseminated not ulcerating nodules (diffuse cutaneous leishmaniasis, DCL). Here we recruited a cohort of CL patients in a newly established leishmaniasis treatment centre (LTC) in Lay Gayint, Northwest Ethiopia, and collected detailed demographic and clinical data. The results of our study show that more males than females present to the LTC to seek diagnosis and treatment. 70.2% of CL patients presented with LCL and 20.8% with MCL. A small number of patients presented with DCL, recidivans CL (a rare form of CL where new lesions appear on the edges of CL scars) or with a combination of different clinical presentations. The duration of illness varied from 1 month to 180 months. Over a third of CL patients had additional suspected CL cases in their household. Despite the majority of CL patients having heard about CL, only a minority knew about its transmission or that it could be treated. Most CL patients lived in areas where environmental factors known to be associated with the transmission of CL were present. This work highlights that CL is an important public health problem in Lay Gayint and emphasises the urgent need for more CL awareness campaigns, better health education and better disease management practices.

## Author summary

Cutaneous leishmaniasis (CL) is a neglected tropical disease caused by species of the *Leishmania* parasite. It causes skin lesions that can be categorised into three main clinical presentations: i) localised cutaneous leishmaniasis (LCL), with one or several ulcerated lesions; ii) mucocutaneous leishmaniasis (MCL) that affects the mucosa of the nose or the mouth; iii) disseminated CL (DCL), that is characterised by widespread nodules. While LCL usually heals spontaneously, MCL and DCL require treatment; and patients often experience relapses. CL leaves disfiguring scars, that can result in societal stigmatisation.

In this study, we recruited a cohort of CL patients in a newly established leishmaniasis treatment centre (LTC) in Lay Gayint, Northwest Ethiopia, and collected detailed demographic and clinical data. We found that most CL patients presented with LCL, followed by MCL, and that more males than females present to the LTC. The duration of illness varied from 1 to 180 months. Over a third of CL patients had additional suspected CL cases in their home. Despite most patients having heard about CL, only a minority knew about its transmission or that it could be treated. This work emphasises that CL is an important public health problem in this area of Ethiopia.

## Introduction

Cutaneous leishmaniasis (CL) is a neglected tropical disease caused by *Leishmania* parasites transmitted by sand fly vectors. It is present in Africa, the Americas, the Eastern Mediterranean, Europe, South-East Asia and the Western Pacific and is endemic in 90 countries [1]. In 2022, 205,662 new cases were reported, with the majority of cases reported from the Eastern Mediterranean [1]. However, due to the absence of CL awareness and reliable reporting systems in many countries, the real number of cases is likely to be much higher. For example, in Africa, out of 19 countries known to be endemic for CL, only 14 had reported cases in 2022 [1]. The disease can cause different clinical manifestations: localised CL (LCL), characterised by one or more ulcerating lesions; mucocutaneous CL (MCL), where the lesions affect the mucosa of the mouth and nose; diffuse CL (DCL), characterised by non-ulcerating nodules; and recidivans CL (RCL), where new lesions appear on the edges of CL scars. Because CL often leaves severe and permanent disfiguring scars, it is frequently associated with discrimination, stigma and substandard living conditions. The diagnosis of CL can be difficult, as it can cause lesions of similar appearance to other skin diseases such as leprosy, bacterial and fungal infections, and eczema [2]. Therefore, cases must be confirmed by identifying parasites in skin scrapings using microscopy or PCR. LCL is the most common form of the disease; it usually heals within one year. However, persistent LCL, MCL, DCL and RCL necessitate treatment and patients still experience frequent relapses [3]. The most commonly used treatments are antimonials; however two recent Cochrane reviews highlighted the low number of well-designed clinical trials that assessed the efficacy of antimonials, as well other treatments used to treat CL, and their long-term effects [4,5].

Over 20 *Leishmania* species can cause CL: in the Old World, CL is mostly caused by *Leishmania (L*.*) tropica*, *L*. *major*, and *L*. *aethiopica*; and in the New World, by *L*. *braziliensis*, *L*. *mexicana and L*. *amazonensis* [6]. In Ethiopia, the majority of CL cases are caused by *L*. *aethiopica*; there have also been reports of CL caused by *L*. *tropica* and *L*. *major* [7]. CL transmission is thought to be mainly zoonotic, with hyraxes being the main reservoir host [8] and *Phebotomus* (P.) *longipes* and *P*. *pedifer* the most common vectors [8,9]. Over 28 million individuals are at risk of CL, primarily in the highlands of Amhara, Oromia, Tigray and the Southern Nations, Nationalities and Peoples’ Region of Ethiopia [10]. While LCL is the most common form of CL in Ethiopia, MCL and DCL are relatively common, but reported percentages of the different clinical forms vary greatly between different studies [11].

Several studies have described the epidemiology of CL in Northern Ethiopia (summarised in [11]). In Tigray, a cross-sectional study performed from November 2011 to April 2012 showed a prevalence of CL of 14% [12]. At the Leishmaniasis Research and Treatment Centre, Gondar, Northern Amhara, a retrospective study showed that 1079 patients were diagnosed with CL over period of 10 years [13]. In these two studies, the different clinical presentations of CL were not identified. In the largest established CL treatment centre in eastern Amhara, in Boru Meda, 888 patients were diagnosed with CL from 2012 to 2018, the majority with LCL (89.2%), 6.9% with MCL and 3.9% with DCL [14]. However, 300km west of Boru Meda, in Lay Gayint where this study took place, no cases had been formally registered by the Amhara Regional Health Bureau until 2019; even though this area had been reported by health professionals to be endemic for CL. Following the establishment of a new Leishmaniasis Treatment Centre (LTC) in Lay Gayint hospital in 2019, and awareness campaigns, we published a preliminary study showing that large numbers of CL patients were identified in this area, with one of two clinical forms: 79.1% with LCL and 20.9% with MCL [15]. We have recently shown that parasites isolated from the lesions of CL patients from the current study were *L*. *aethiopica*; we did not identify any individual genetic variants significantly associated with disease presentation [16]. Little is known about the demographic characteristics of patients presenting with the different forms of CL in this area.

The aim of this study was to recruit a cohort of CL patients in Lay Gayint to provide detailed clinical description of the different forms of CL; as well as obtain detailed documentation of living habits, family history of CL, and environmental conditions and investigate how these factors may be associated with different CL presentations.

## Materials and methods

### Ethical approvals

This study was approved by the Research and Ethical Review Committee of the College of Science, Bahir Dar University (RCSVD 002/2011 EC), the National Research Ethics Review Committee of the Ministry of Science and Higher Education of Ethiopia (ref. No MoSHE/RD/ 14.1/10112/2020) and Imperial College Research Ethics Committee (ICREC 18IC4593). Informed written consent was obtained from each adult and from the parent/guardian for each child.

### Study area

This study was carried out in Nefas Mewcha Hospital, a primary hospital in Lay Gayint District, Northwest Ethiopia. Lay Gayint is found in the South Gondar administrative zone of the Amhara National Regional State (11° 50’ 59” N latitude and 38° 22’ 0” E longitude). The district of Lay Gayint has 9 health centres, 43 health posts, and 1 primary hospital, that are providing health care for an estimated population of 211,475 (projected from the latest official census in 2007). The district covers an area of about 1,522.4 km^2^, with a population density of 163.6 people/km^2^. The topography of the district is dominated by chains of mountains, hills, and valleys extending from the Tekeze river (1494m above sea level) to the Guna Mountain Summit (3991m above sea level). The annual mean minimum and maximum temperatures range from 8°C to 29°C; and the average annual rainfall of the district is 898.3mm.

### Cutaneous leishmaniasis patient recruitment

Following awareness campaigns [15], health extension workers identified individuals with potential CL lesions in their respective catchment areas and referred them to the LTC, in Nefas Mewcha Hospital. Some individuals with skin lesions also came after they heard about the LTC. All individuals were seen by a dermatologist who triaged them based on the clinical appearance of the lesions: a parasitological diagnosis was performed on potential CL patients and all other patients were referred for further tests.

### Diagnosis of CL

A parasitological diagnosis was used to confirm CL: a skin scraping was collected from the edge of the active lesion using a sterile scalpel. The scraping was smeared on a glass slide and stained with 10% Giemsa stain to identify and count the number of amastigotes by microscopy as described in [17]. The same grading system as that described in [17] was used to grade the number of amastigotes per slide. If the slide was negative, but the lesions had all the clinical features of CL as defined by The Guidelines for Diagnosis, Treatment and Prevention of Leishmaniasis in Ethiopia [2] (a clinically suspicious lesion is defined as a skin nodule or ulcer with a raised edge appearing on someone who lives in an area known to be endemic for CL or visited such an area in the last 2 years), the patient was still considered to be a CL patient. Confirmed CL cases were treated with sodium stibogluconate i.m. (20 mg/kg/day) for 28 days, as described in the Guidelines for Diagnosis, Treatment and Prevention of leishmaniasis in Ethiopia.

### Collection of demographic and clinical data

A standardised interviewer-administrated questionnaire was used to collect socio-demographic and clinical information. For CL patients < 18 years old, their parent or guardian was asked to answer the questions. The duration of illness was defined as the time (in months) since the first lesion appeared. The number of lesions was counted by the interviewer and varied from 1 to >5 lesions. Body mass index (BMI) was measured by dividing body weight (kg) by the square of height (m).

### Knowledge about CL

To evaluate the knowledge of adult patients about CL, the following three questions were asked:

- Had they heard about CL, locally named as “kuncher”?
- Did they know how the disease is transmitted, and if so, how?
- Did they know if the disease can be treated, and if so where?

### Statistical analysis

Data were evaluated for statistical differences as specified in the legend of each table and figure. Fisher’s exact test was used to test for associations between age or sex and CL type, and for a difference in the distribution of parasite gradings between adults and children. Mann-Whitney and Kruskal-Wallis tests were used to assess differences in illness duration and lesion number by CL type using Prism 10. Spearman’s rank correlation coefficient, *ρ*, was used to assess correlation between illness duration and lesion number. Differences were considered statistically significant at *p*<0.05. * = p<0.05, ** = p<0.01, *** = p<0.001 and **** = p<0.0001. Unless otherwise stated, summary statistics given are medians followed by interquartile range (IQR) in square brackets.

## Results

### Recruitment

**Fig 1:**
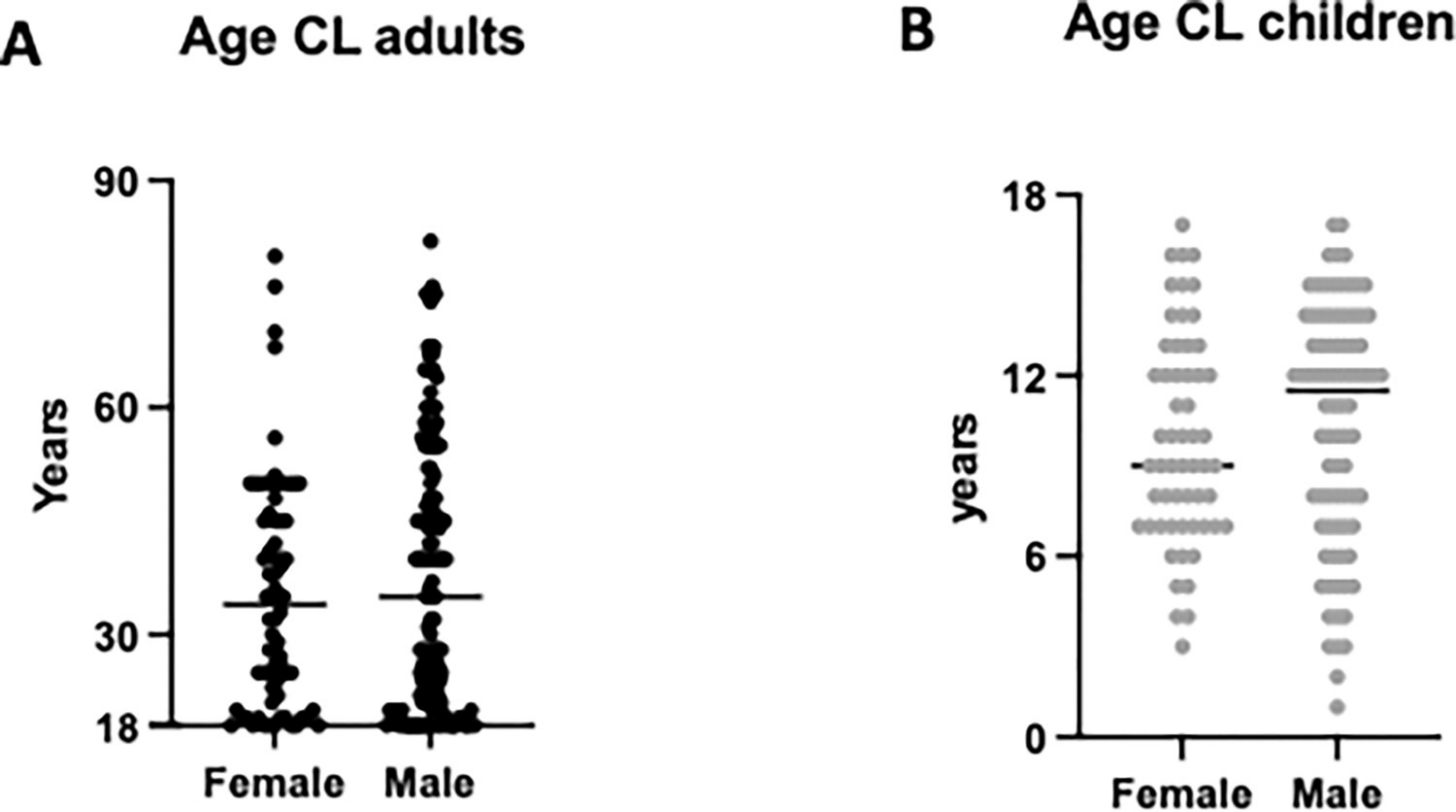
Ages of CL patients. (A) Ages of adult female (n = 72) and male (n = 135) CL patients. B. Ages of child female (n = 57) and male (n = 82) CL patients. Statistical differences were determined using a Mann-Whitney test. The straight line represents the median. The differences were not significant.

**Fig 2:**
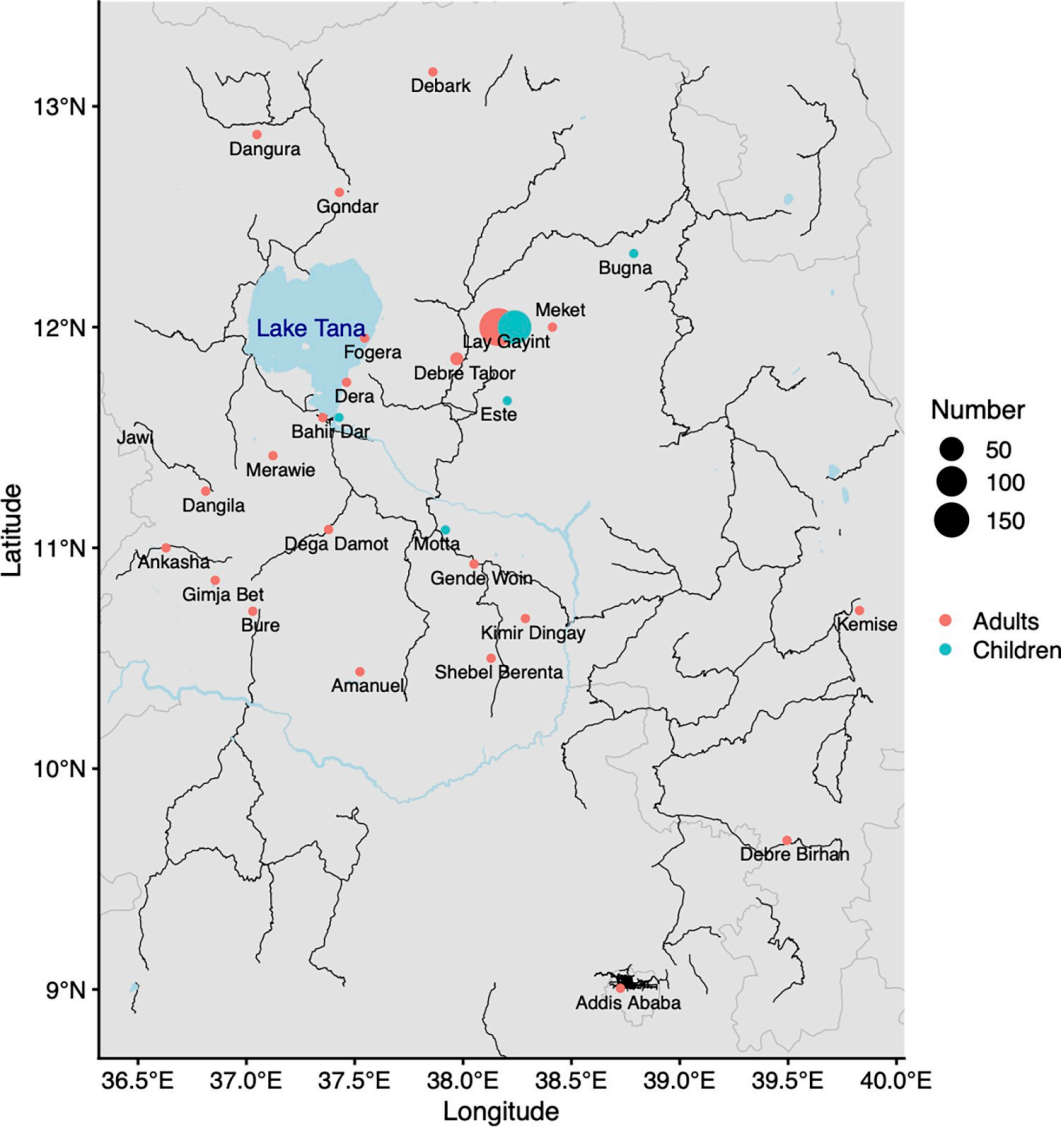
Map of the permanent places of residence of the recruited CL patients in Northern Ethiopia. Red dots represent adult CL patients, green dots child CL patients; and dot size represents the number of CL patients coming from each place of residence. Black lines show roads, grey lines show state boundaries. Map created in R v4.0.5 [35] using a base map of the region from Open Street Map [36] https://www.openstreetmap.org/#map=8/11.084/38.200.

In this study, we recruited 346 CL patients in a newly established Leishmaniasis Treatment Centre (LTC), in Nefas Mewcha Hospital, Lay Gayint. The recruitment took place from January 2019 to September 2022. 207 CL patients were adults and 139 were children. 72 adult patients were female with a median age of 35 [21.3–45.8] and 135 were male with a median age of 35 [21–52] (p = 0.4542, Fig 1A). 57 child patients were female with a median age of 9 [7–12.5] and 82 were male with a median age of 11.5 [7–14] (p = 0.3353) (Fig 1B). Adult patients came from 23, and children from 5, different districts (Fig 2 and S1 Table). Most CL patients came from Lay Gayint (182 adult [87.9%] and 135 children [97.1%], S1 Table).

### Clinical forms of CL

**Fig 3:**
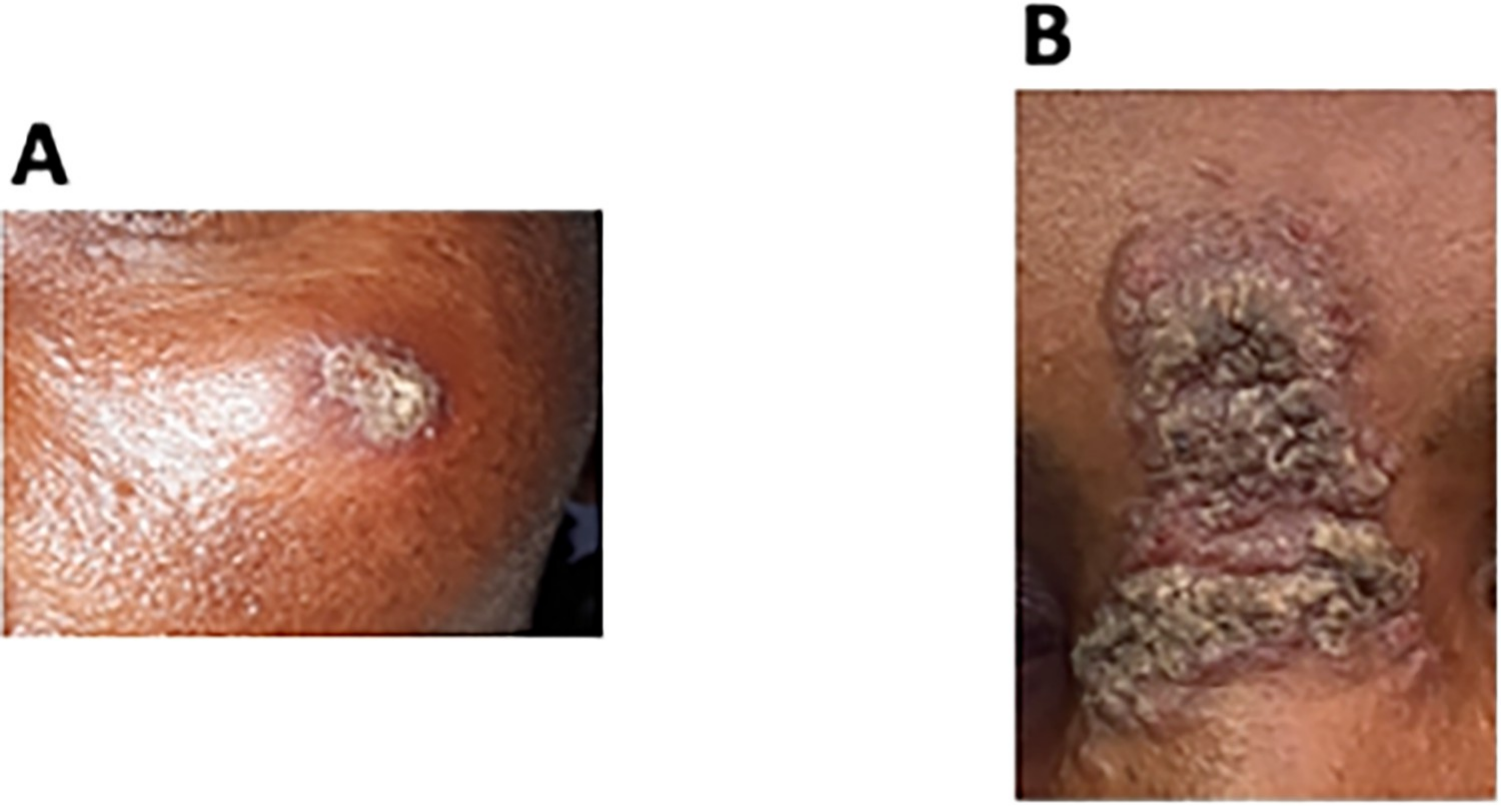
Examples of contained and spreading LCL lesions. **A.** Contained lesion, with a distinct border around the lesion (contained LCL, C LCL). **B.** Spreading lesion, without clear edges (spreading LCL, S LCL).

CL patients presented with different clinical forms: localised CL (LCL), mucocutaneous CL (MCL), diffuse CL (DCL), and recidivans (RCL). LCL patients were further divided into two groups: those presenting with a well-defined contained lesion, with a distinct border around the lesion (contained LCL, C LCL) (Fig 3A) and those presenting with a lesion that did not have clear edges and was spreading (spreading LCL, S LCL) (Fig 3B). Some CL patients presented with multiple clinical forms of CL (multiple CL, MCL + C LCL, MCL + S LCL, S LCL + C LCL and DCL + MCL, Table 1).

**Table 1:** Frequency of different clinical forms of CL.

Clinical presentations	Adults n (%)		Children n (%)	
**LCL**	145 (70)		98 (70.5)	
	**C LCL**	**S LCL**	**C LCL**	**S LCL**
	105 (72.4)	40 (27.6)	72 (73.5)	26 (26.5)
**MCL**	49 (23.7)		23 (16.5)	
**DCL**	4 (1.9)		1 (0.8)	
**RCL**	1 (0.5)		5 (3.6)	
**Multiple CL** *	8 (3.9)		12 (8.6)	

* 12 patients with MCL + C LCL, 4 with MCL + S LCL, 3 with S LCL + C LCL and 1 with DCL + MCL

Results presented in Table 1 show that the majority of adult and child CL patients presented with LCL (70% and 70.5%, respectively). There was no significant difference in the distribution of forms of CL between males and females, for either adults (p = 0.2583) or children (p = 0.3247). Amongst adult and child LCL patients, there were more C LCL than S LCL (Table 1). 23.7% of adults and 16.5% of children presented with MCL (Table 1); 1.9% and 0.8% with DCL and 0.5% and 3.6% with recidivans CL. Eight adults and 12 children presented with multiple CL (Table 1).

Amongst adults, the most represented age group of CL patients was 18–29 years. Amongst children, there was a similar number of CL patients in the 0–9 (n = 62) and the 10–17 (n = 77) age groups (S2 Table). The most common form of CL was C LCL for all ages and sexes (S2 Table). There was no association between age and form of CL for either adults (p = 0.2237) or children (p = 0.1475).

### CL diagnosis (parasitological/clinical)

Parasitological diagnosis was used to confirm CL: 157 adults and 107 children tested positive by microscopy. The distribution of gradings of the number of amastigotes per slide is shown in Table 2. Most gradings were 1+ for both adults and children. There was no significant difference in the distribution of parasitological gradings between adults and children (p = 0.8142). Fifty adults and 26 children tested negative but based on the appearance of their lesion(s) were considered to have CL by the dermatologist.

**Table 2:** Grading of amastigotes.

	Adults	Children
**1+**	84	50
**2+**	36	31
**3+**	15	12
**4+**	13	7
**5+**	6	3
**6+**	3	4

The grading was performed as described in Materials and Methods.

### Duration of illness

**Fig 4:**
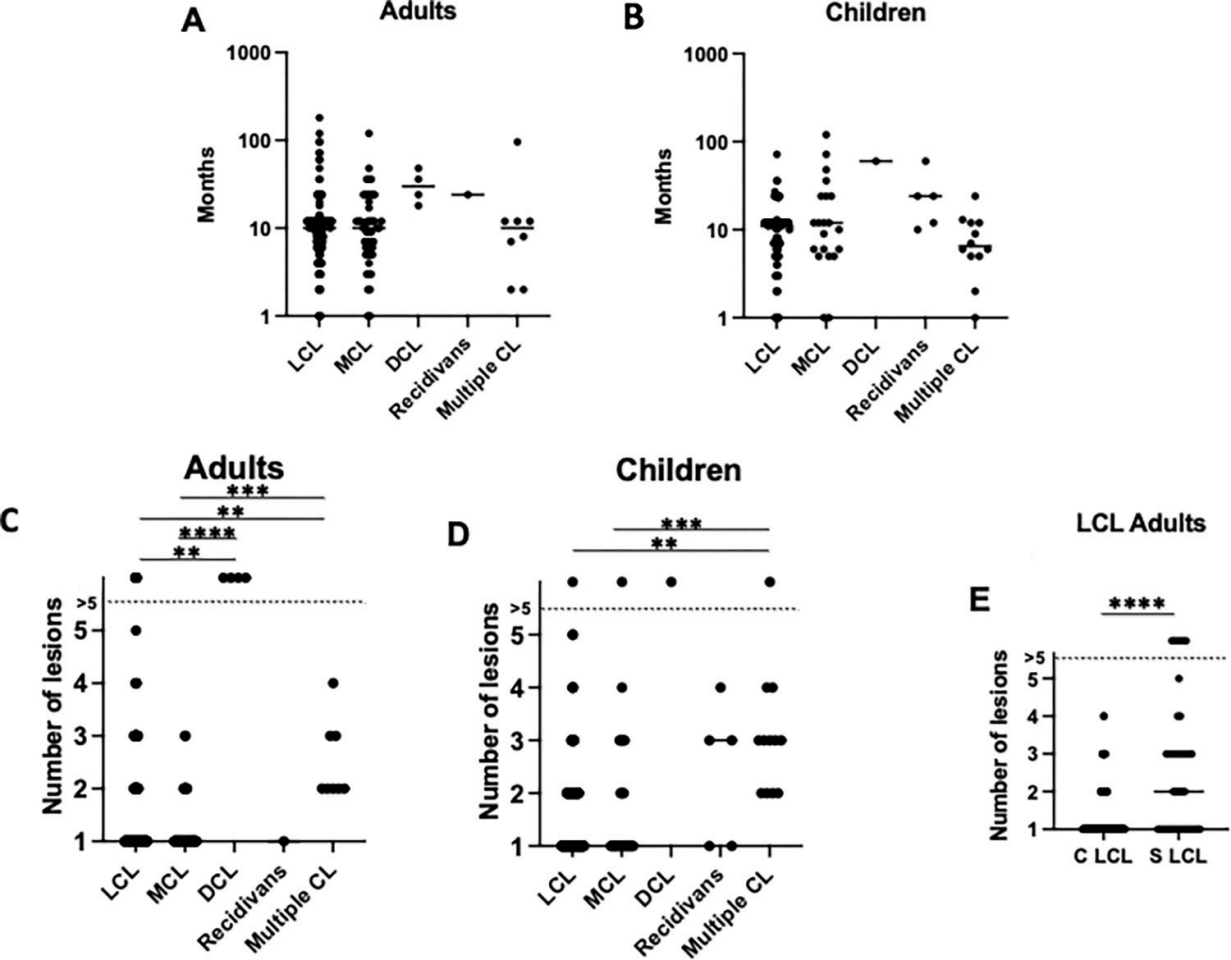
Duration of illness and number of lesions. **A.** Duration of illness in months for each clinical presentation in adult CL patients (LCL: n = 144, MCL: n = 49, DCL: n = 4, recidivans: n = 1, multiple CL: n = 8); this information was missing for 1 LCL patient. **B.** Duration of illness in months for each clinical presentation in child CL patients (LCL: n = 94, MCL: n = 21, DCL: n = 1, recidivans: n = 5, multiple CL: n = 12); this information was missing for 4 LCL and 2 MCL patients. **C.** Number of lesions for each clinical presentation in adult CL patients (LCL: n = 145, MCL: n = 49, DCL: n = 4, recidivans: n = 1, multiple CL: n = 8). **D.** Number of lesions for each clinical presentation in child CL patients (LCL: n = 98, MCL: n = 23, DCL: n = 1, recidivans: n = 5, multiple CL: n = 12). **E.** Number of lesions in adult S (n = 105) and S (n = 40) LCL patients. In Fig 4A, 4B, 4C and 4D, statistical differences between the five different clinical presentations of CL were measured by Kruskal-Wallis test and the multiple comparison between each clinical presentation using Dunn’s multiple comparison test. In Fig 4E, statistical difference between S and C LCL was determined using a Mann-Whitney test. The straight line represents the median. Only significant differences were annotated, in the absence of *, the differences were not significant.

In adults, the duration of illness varied from 1 month to 180 months (Fig 4A and S3 Table) and in children from 1–120 months (Fig 4B and S3 Table). There were no significant differences in duration of illness between the different forms of CL in adult and child patients (p = 0.0628 and p = 0.0518, Fig 4A and 4B); or between adult (p = 0.1084) and child (p = 0.8601) C LCL and S LCL patients (S3 Table).

### Number of lesions

In adult and child patients, the numbers of lesions varied from 1 to >5 (Fig 4C), and the majority (60.4%) of CL patients presented with one lesion. The highest numbers of lesions were identified in patients with DCL (Fig 4C and 4D and S4 Table).

There was a significant difference in the numbers of lesions between the different CL presentations in adults (Fig 4C and S4 Table), with the numbers of lesions in LCL patients being lower than in DCL (p = 0.0012) and multiple CL (p = 0.0077) patients and the numbers of lesions in patients with MCL being lower than in DCL (p<0.0001) and multiple CL (p = 0.0003) patients.

In children, there was also a significant difference in the numbers of lesions between the different CL presentations (Fig 4D and S4 Table), with the numbers of lesions in patients with multiple CL being higher than in LCL (p = 0.0003) and MCL (p = 0.0046) patients.

The numbers of lesions were significantly higher in S LCL than C LCL in adults (Fig 4E, p<0.0001), but not in children (p = 0.1006).

Of note, for adult and child CL patients, there were no significant differences in duration of illness (p = 0.5350 and p = 0.5419) or number of lesions (p = 0.9912 and p = 0.2673) between those who had a positive parasitological diagnosis and those who had a negative parasitological diagnosis.

There were weak positive correlations between the number of lesions and the duration of illness in adults and in children (*ρ* = 0.246, p = 0.0004 and *ρ* = 0.241, p = 0.0051, Table 3). When stratified according to the different clinical presentations, there was a weak positive correlation for adult patients with S LCL (*ρ* = 0.382, p = 0.0165) and for child patients with C LCL and MCL (*ρ* = 0.266, p = 0.0269 and *ρ* = 0.640, p = 0.0018) (Table 3).

**Table 3:** Correlations between the number of lesions and the duration of illness.

	Rank correlation coefficient	p value^‡^
**Adults**		
**CL (n = 206)**	0.246	0.0004
**C LCL (n = 105)**	0.144	0.1460
**S LCL (n = 39)**	0.382	0.0165
**MCL (n = 49)**	0.162	0.2665
**Children**		
**CL (n = 133)**	0.241	0.0051
**C LCL (n = 69)**	0.266	0.0269
**S LCL (n = 25)**	0.010	0.9619
**MCL (n = 21)**	0.640	0.0018

^‡^Spearman test

### Location of lesions

The majority of lesions were located on the face, with cheek and nose being the most affected areas (S5 Table). A small number of patients had lesions on their ear, hand, thigh, shoulder or neck, or in multiple locations (S5 Table).

### CL patients’ occupations and education

There were four main occupations in adult CL patients (S6 Table): farmer, government employee, student and merchant. The majority of patients were farmers (72%), followed by students (20.3%), government employees (5.8%) and merchants (1.9%).

The levels of education were also assessed and as shown in S6 Table, the majority of CL patients were illiterate (58.4%). Students recruited in this study were studying at primary, secondary school, or college and above. Most children were in primary or secondary school (S7 Table).

### BMI

The median BMIs for female (20.6 [18.5–22.5]) and male (21.0 [19.5–22.9]) adults were similar (p = 0.0936) and there was no statistically significant difference in BMI between the different clinical forms (C LCL = 20.9 [19.3–22.8]; S LCL = 21.1 [19.1–22.9]; MCL = 20.0 [19.0–22.2]; DCL = 24.8 [21.6–28.7]; recidivans CL = 19.6 [19.6–19.6]; multiple CL = 21.2 [18.7–25.9; p = 0.1963).

### History of CL in the household

207 adult CL patients were asked if someone else in the household was potentially presenting with CL lesions, as indicated by a health extension worker: 26 (12.6%) said that health extension workers had identified potential CL lesions (see Table 4 for details). For children, 52 guardians or parents were asked: 18 (34.6%) mentioned about a family member with suspected CL lesions (Table 4).

**Table 4:** Potential other CL cases in the households of diagnosed CL patients.

**ADULTS**						
**Diagnosed CL patients**			**Potential CL cases**			
**Age range of each patient**	**CL**	**Duration of illness**	**n**	**Age range of each patient**	**Duration of illness**	**Treatment**
16–20	S LCL	12m	2	36-40/21-25	12m	No
16–20	S LCL	36m	1	56–60	12m	Yes, unknown
50–56	C LCL	7m	1	56–60	5m	No
36–40	MCL	20m	1	11–15	3m	No
31–35	C LCL	10m	1	6–10	24m	Yes, traditional
46–50	S LCL	12m	1	26–30	12m	Yes, traditional
21–25	C LCL	3m	1	0–5	9m	No
26–30	C LCL	4m	1	26–30	2m	No
35–40	MCL	12m	1	11–15	24m	No
16–20	MCL	7m	1	26–30	6m	No
56–60	MCL	120m	3	16-20/6-10/6-10	12m	Yes, traditional
56–60	C LCL	12m	1	16–20	12m	Yes, traditional
46–50	MCL	12m	1	26–30	1m	Yes, unknown
36–40	Mixed	2m	1	31–35	2m	Yes, SSG
61–65	S LCL	5m	1	0–5	5m	No
56–60	C LCL	4m	1	50–56	7m	No
41–45	C LCL	1m	2	16-20/16-20	36m	No
66–70	S LCL	7m	1	0–5	6m	No
46–50	C LCL	7m	1	6–10	7m	No
61–65	C LCL	10m	1	50–56	12m	Yes, SSG
18–20	MCL	2m	1	36–40	12m	Yes, SSG
26–30	MCL	2m	1	26–30	4m	Yes, traditional
71–75	MCL	4m	2	36-40/50-56	12m	No
26–30	C LCL	12m	1	0–5	3m	No
21–25	C LCL	24m	1	16–20	12m	No
31–35	C LCL	4m	1	11–15	4m	No
**CHILDREN**						
**Diagnosed CL patients**			**Potential CL patients**			
**Age range of each patient**	**CL**	**Duration of illness**	**n**	**Age range of each patient**	**Duration of illness**	**Treatment**
6–10	MCL	1m	1	0–5	12m	No
6–10	C LCL	7m	1	46–50	7m	No
11–15	C LCL	12m	1	6–10	24m	No
16–20	MCL	5m	1	11–15	9m	No
0–5	S LCL	12m	1	6–10	1m	No
11–15	C LCL	12m	1	6–10	6m	No
6–10	S LCL	3m	1	11–15	12m	No
6–10	C LCL	7m	1	15	24m	No
0–5	MCL	12m	1	12	12m	Yes, traditional
6–10	MCL	6m	1	14	12m	No
11–15	C LCL	12m	1	9	6m	No
6–10	S LCL	7m	1	5	7m	No
11–15	Mixed	9m	1	16	5m	No
11–15	C LCL	24m	1	54	12m	No
6–10	RCL	60m	1	14	24m	No
11–15	C LCL	12m	1	9	24m	No
11–15	C LCL	24m	1	2	48m	No
0–5	S LCL	5m	1	10	24m	Yes, traditional

Their age, the duration of illness and the treatment were also recorded (Table 4). The majority had not received treatment yet.

### Knowledge about CL

Most CL patients (66.7%) had heard about CL, mainly from a member of their family or a friend (S8 Table). A small percentage had heard about CL from a health facility or from school. Only 3.4% knew that CL is transmitted by an insect and 93.2% did not know that CL lesions can be treated (S8 Table).

### Environmental factors

A total of 304 individuals (CL patients and parents or guardians of child patients) were asked about environmental factors known to be associated with the transmission of CL. The first question was about the presence of domestic animals such as dogs, goats, sheep, donkeys, cows, horses, cats, and/or chickens, as well as hyraxes, which are the main host reservoir for *L*. *aethiopica*. All CL patients had seen all these animals where they live or work, except for 23 CL patients, who had seen all the domestic animals but no hyraxes.

Next, they were asked if they live or work close to places where sand flies are known to breed, as well as places where hyraxes live such as caves, gorges, animal burrows, and or/ rock piles. All were present where the CL patients live or work.

Sand flies feed on the nectar, honeydew, and sap of certain trees, mainly *Acacia* and *Balanites* species. CL patients were asked to name the main trees present around the places where they live and work. Most CL patients (n = 207) lived in the vicinity of *Acacia* and/or *Balanites*. Some other trees were also mentioned such as eucalyptus and juniper. 63 CL patients did not mention *Acacia* or *Balanites* but did mention tree species that are not associated with sand flies.

All houses where the CL patients lived had thatched grass wall, with cracks. All but one female and one male slept inside the house. 30 CL patients occasionally used bed nets, and none used an insect repellent.

## Discussion

Our paper is the most detailed socio-demographic and epidemiological study to date of CL patients presenting in a recently established Leishmaniasis Treatment Centre (LTC), in Nefas Mewcha, Lay Gayint. CL in this area was poorly characterised. The District Health Office had described an outbreak of CL in Lay Gayint in 2009 and health professionals knew of CL cases from this part of Amhara. However, these had never been officially recorded by the Amhara Regional Health Bureau until 2019. We have recently published a study, describing the establishment of a new LTC in Nefas Mewcha in 2019 and reporting the retrospective data of a large number of CL patients presenting to this LTC [15]. This study highlighted that CL is a major health problem in this area.

In the current study, we recruited 207 adult and 139 child CL patients. Fifty adults and 26 children tested negative by parasitological diagnosis. Since PCR is not available in this setting, these patients were diagnosed as CL patients by highly experienced clinical staff based on the appearance of their lesions, as described in [2]. We can however not demonstrate that all these individuals were indeed CL patients and therefore cannot exclude possible biases in our analyses.

This study took place over a 33-month period. The recruitment and follow-up of patients were severely hampered by the COVID-19 pandemic and the civil war in Ethiopia. Due to these problematic recruitment conditions, patients were recruited as they came to the LTC. It is therefore not possible to infer the prevalence or incidence rate of active CL in adults or children in this area from these data.

As previously described, there were more adult and child males than females presenting with CL [14,15,18,19]. In our study this did not appear to be due to a difference in risk between males and females associated with different occupations as the majority of adult CL patients were farmers (who are at high risk of being bitten as they spend long periods of time working outside) and the proportion who were farmers among males and females was very similar (72.6% vs 70.9%, p = 0.5020). It is possible that men spend more time outside, as women oversee the cooking and might therefore spend more time indoors, and that this could explain why more males present with CL than females. However, it is also possible that men are more likely to seek diagnosis and treatment than women, at least in part because of the long and difficult journey to travel to the LTC. For some patients, the LTC was several days away from their villages.

As shown previously [15] and in agreement with previous studies in Ethiopia [11,13,20], the highest number of CL patients recruited were amongst children and young adults in the 18–29 age range. Due to the nature of recruitment, it is not possible to ascertain that this is representative of the age distribution of CL in this population. However, it has been shown that previous *Leishmania* infection can confer a high degree of protection against subsequent infection: leishmanization, where individuals are infected with virulent *L*. *major* parasites, has been shown to protect against subsequent natural infection in highly endemic areas [21]. It is therefore possible that in our cohort of CL patients, there are more younger individuals with active CL lesions, as older adults are likely to have been infected earlier in life and have become immune to reinfection.

LCL is the most common clinical presentation of CL worldwide [22], and Northern Ethiopia is no exception [11,23]. Indeed, in our study in Lay Gayint, over 65% of adults and children presented with LCL. Our cohort of patients presenting with LCL was subdivided into two separate groups, those with a well-defined contained lesion (C LCL) that had a clear border and those presenting with a lesion that did not have distinct edges and was spreading (S LCL). The majority of LCL patients presented with C LCL, but almost a third with S LCL. It is tempting to speculate that nonhealing C LCL might become S LCL over time. However, we did not observe any difference in duration of illness. Since this was a cross-sectional study and none of the patients were followed, we do not know the fate of C LCL lesions. A longitudinal follow-up from early lesions would allow for a better description of the clinical evolution of self-healing and spreading persistent lesions. MCL is usually quite rare, though the percentages of MCL reported by different studies vary greatly. In our cohort, the percentage of CL patients presenting with MCL was relatively high, 23.7% in adult patients and 16.5% in child patients. A study performed in Gondar, Northern Amhara, also showed a high percentage (42.7%) of patients presenting with MCL to the Leishmaniasis Research and Treatment Centre [23]. A small number of patients in our study also presented with different clinical presentations, with DCL or RCL. Since these forms of CL can be severely disfiguring, some of these patients hide their lesions and do not dare to seek diagnosis. A small percentage of CL patients presented with multiple CL. Disseminated leishmaniasis (DL) is a severe form of cutaneous leishmaniasis with several polymorphic lesions [24], it usually is associated with *L*. *braziliensis* and presents with >10 lesions. It is therefore unlikely that the patients with multiple CL recruited in the present study were DL patients as they presented with considerably less lesions (less than 4 lesions, except for one patient who had 6 lesions). However, we cannot exclude that their clinical symptoms might evolve into more severe presentations such as DL with time.

Of note, the duration of illness was longer in adult (n = 4) and child (n = 1) DCL patients, this is not unexpected as DCL is known to be progressive and nonhealing ([2,25,26]). Care should be taken with these results as only a small number of DCL patients were recruited in our study.

A considerable percentage of CL patients indicated that a health extension worker had identified other members of their family with potential CL lesions. This indicates that infection with *Leishmania* is not necessarily only associated with the occupation of the patients and could occur inside or close to the house [27,28].

Most CL patients in our study had heard about CL, mainly from a member of their family, but very few from health facilities or even schools. Importantly, most did not know how it is transmitted and that it can be treated. This is in line with findings from other studies, where patients or individuals living in endemic areas know about the disease, but little about its transmission and treatments [29–32]. Better knowledge about how the disease is transmitted and the use of bed nets are likely to reduce CL transmission by sand flies. Indeed, it has been shown that systematic use of bed nets, in conjunction with indoor residual spraying, resulted in reduced incidence of CL in Mali [33]. The poor knowledge of the population studied here about treatment is likely to contribute to the long duration of illness and the severity of some of the lesions that were observed in this study. These often result in permanent disfiguration that can lead to social stigmatisation and a mental health burden [2]. Close follow-up of lesions to identify persistent nonhealing lesions that might benefit from early treatment is likely to both improve clinical outcomes [34] and reduce the risk of onward transmission.

The lack of knowledge about the extent of CL in this area, and in Ethiopia in general, is impeding effective control and prevention strategies. This work reinforces that CL is a major public health problem in Lay Gayint and emphasises the urgent need for more CL awareness campaigns, better health education and better disease management practices.

## Supporting information

S1 Table Permanent place of residence of CL patients. Number of adult and child CL patients presenting to the Leishmaniasis Treatment Center in Nefas Mewch and their permanent place of residence.
(DOCX)

S2 Table Frequency of different forms of CL by age group. Number of Child and adult CL patients presenting with different clinical presentation per age group. CL = cutaneous leishmaniasis; C LCL = contained localised CL; S LCL: spreading localised CL; MCL = mucocutaneous CL; DCL = diffuse CL; RCL = recidivans CL.
(DOCX)

S3 Table Duration of illness (in months) by form of CL. The durations of illness in adult CL patients were recorded for 144 LCL, 49 MCL, 4 DCL, 1 recidivans CL and 8 multiple CL; this information was missing for 1 LCL patient. For child CL patients, it was recorded for 94 LCL, 21 MCL, 1 DCL, 5 recidivans CL and 12 multiple CL; this information was missing for 4 LCL and 2 MCL patients. For the comparison between C LCL and S LCL, the durations of illness were recorded for 105 C LCL and 39 S LCL patients in adults (this information was missing for 1 S LCL patient) and for 69 C LCL (this information was missing for 3 C LCL patients) and 25 S LCL patients (this information was missing for 1 S LCL patient) in children. CL = cutaneous leishmaniasis; C LCL = contained localised CL; S LCL: spreading localised CL; MCL = mucocutaneous CL; DCL = diffuse CL; RCL = recidivans CL. *Statistical difference measured by Kruskal-Wallis. ^#^ Statistical difference measured by Mann-Whitney.
(DOCX)

S4 Table Number of lesions by CL type. The numbers of lesions in adult CL patients were recorded for 145 LCL, 49 MCL, 4 DCL, 1 recidivans CL and 8 multiple CL. For child CL patients, it was recorded for 98 LCL, 23 MCL, 1 DCL, 5 recidivans CL and 12 multiple CL. For the comparison between C LCL and S LCL, the numbers of lesions were recorded for 105 C LCL and 40 S LCL in adults and for 72 C LCL and 26 S LCL in children. CL = cutaneous leishmaniasis; C LCL = contained localised CL; S LCL: spreading localised CL; MCL = mucocutaneous CL; DCL = diffuse CL; RCL = recidivans CL.*Statistical difference measured by Kruskal-Wallis. ^#^ Statistical difference measured by Mann-Whitney.
(DOCX)

S5 Table Location of lesions. Multiple locations: lesion on at least 2 of the following sites: face, ear, hand, neck, back, chest or leg.
(DOCX)

S6 Table Occupation/education of adult CL patients. Number of adult CL patients and their occupation and their education levels. *Students attending Orthodox Church schools. na = not applicable
(DOCX)

S7 Table Education of child CL patients. Number of child adult patients and their education levels.
(DOCX)

S8 Table Knowledge about CL, transmission and treatment. Number of patients who knew about CL, its transmission and treatment. *2 CL patients gave incorrect answers: transmission by bats or by direct contact. na = not applicable.
(DOCX)
