# Supplementary material for: Demographic characteristics and clinical features of patients presenting with different forms of cutaneous leishmaniasis, in Lay Gayint, Northern Ethiopia
Source: PLoS Negl Trop Dis. 2024 Aug 15;18(8):e0012409. doi: 10.1371/journal.pntd.0012409 (PMC11349221; doi:10.1371/journal.pntd.0012409)
Supplement: S1 Table — Number of adult and child CL patients presenting to the Leishmaniasis Treatment Center in Nefas Mewch and their permanent place of residence. (DOCX) [file pntd.0012409.s001.docx]

**S1 Table: permanent place of residence of CL patients**

| District/**city** | Adults | Children |
| --- | --- | --- |
|  |  |  |
| **Addis Ababa** | 1 | 0 |
| Amanuel | 1 | 0 |
| Ankasha | 1 | 0 |
| **Bahir Dar** | 1 | 1 |
| Bure | 1 | 0 |
| Bugna | 0 | 1 |
| Dangila | 1 | 0 |
| Dangura | 1 | 0 |
| Debark | 1 | 0 |
| Debre Birhan | 1 | 0 |
| Debre Tabor | 4 | 0 |
| Dega Damot | 1 | 0 |
| Dera | 1 | 0 |
| Este | 0 | 1 |
| Fogera | 1 | 0 |
| Gimja Bet | 1 | 0 |
| Gende Woin | 1 | 0 |
| Gondar | 1 | 0 |
| Jawi | 1 | 0 |
| Kemise | 1 | 0 |
| Kimir Dingay | 1 | 0 |
| Lay Gayint | 182 | 135 |
| Meket | 1 | 0 |
| Merawie | 1 | 0 |
| Motta | 0 | 1 |
| Shebel Berenta | 1 | 0 |
