# Supplementary material for: Demographic characteristics and clinical features of patients presenting with different forms of cutaneous leishmaniasis, in Lay Gayint, Northern Ethiopia
Source: PLoS Negl Trop Dis. 2024 Aug 15;18(8):e0012409. doi: 10.1371/journal.pntd.0012409 (PMC11349221; doi:10.1371/journal.pntd.0012409)
Supplement: S2 Table — Number of Child and adult CL patients presenting with different clinical presentation per age group. CL = cutaneous leishmaniasis; C LCL = contained localised CL; S LCL: spreading localised CL; MCL = mucocutaneous CL; DCL = diffuse CL; RCL = recidivans CL. (DOCX) [file pntd.0012409.s002.docx]

**S2 Table: Frequency of different forms of CL by age group**

| **Children** | **C LCL**  **(n)** | **S LCL**  **(n)** | **MCL**  **(n)** | **DCL**  **(n)** | **RCL**  **(n)** | **Multiple CL (n)** | **Total**  **(n)** |
| --- | --- | --- | --- | --- | --- | --- | --- |
| 0-9 | 27 | 16 | 10 | 0 | 4 | 5 | 62 |
| 10-17 | 45 | 10 | 13 | 1 | 1 | 7 | 77 |
| **Adults** | **C LCL**  **(n)** | **S LCL**  **(n)** | **MCL**  **(n)** | **DCL**  **(n)** | **RCL**  **(n)** | **Multiple CL (n)** | **Total**  **(n)** |
| 18-29 | 45 | 23 | 19 | 3 | 0 | 2 | 92 |
| 30-39 | 20 | 5 | 2 | 0 | 1 | 0 | 28 |
| 40-49 | 16 | 4 | 15 | 1 | 0 | 3 | 39 |
| 50-59 | 17 | 4 | 7 | 0 | 0 | 0 | 28 |
| 60-69 | 9 | 4 | 2 | 0 | 0 | 1 | 16 |
| 70-79 | 3 | 0 | 3 | 0 | 0 | 1 | 7 |
| 80-89 | 1 | 0 | 1 | 0 | 0 | 0 | 2 |
