# Supplementary material for: Demographic characteristics and clinical features of patients presenting with different forms of cutaneous leishmaniasis, in Lay Gayint, Northern Ethiopia
Source: PLoS Negl Trop Dis. 2024 Aug 15;18(8):e0012409. doi: 10.1371/journal.pntd.0012409 (PMC11349221; doi:10.1371/journal.pntd.0012409)
Supplement: S3 Table — The durations of illness in adult CL patients were recorded for 144 LCL, 49 MCL, 4 DCL, 1 recidivans CL and 8 multiple CL; this information was missing for 1 LCL patient. For child CL patients, it was recorded for 94 LCL, 21 MCL, 1 DCL, 5 recidivans CL and 12 multiple CL; this information was missing for 4 LCL and 2 MCL patients. For the comparison between C LCL and S LCL, the durations of illness were recorded for 105 C LCL and 39 S LCL patients in adults (this information was missing for 1 S LCL patient) and for 69 C LCL (this information was missing for 3 C LCL patients) and 25 S LCL patients (this information was missing for 1 S LCL patient) in children. CL = cutaneous leishmaniasis; C LCL = contained localised CL; S LCL: spreading localised CL; MCL = mucocutaneous CL; DCL = diffuse CL; RCL = recidivans CL. *Statistical difference measured by Kruskal-Wallis. # Statistical difference measured by Mann-Whitney. (DOCX) [file pntd.0012409.s003.docx]

**S3 Table: Duration of illness (in months) by form of CL**

|  | **LCL** | **MCL** | **DCL** | **Recidivans** | **Multiple CL** | **p values*** |
| --- | --- | --- | --- | --- | --- | --- |
| **Adult** | 10 [6-12] | 10 [5.5-18.5] | 30 [19.5-45] | 24 | 10 [3.3-12] | 0.0628 |
| **Children** | 11 [5.8-12] | 12 [5.5-24] | 60 | 24 [11-24] | 6.5 [5-12] | 0.0518 |
|  | **C LCL** | **S LCL** | **p values^#^** |  |  |  |
| **Adult** | 9 [6-12] | 12 [7-18] | 0.1084 |  |  |  |
| Children | 11 [5-12] | 11 [7-12] | 0.8601 |  |  |  |

The durations of illness in adult CL patients were recorded for 144 LCL, 49 MCL, 4 DCL, 1 recidivans CL and 8 multiple CL; this information was missing for 1 LCL patient. For child CL patients, it was recorded for 94 LCL, 21 MCL, 1 DCL, 5 recidivans CL and 12 multiple CL; this information was missing for 4 LCL and 2 MCL patients. For the comparison between C LCL and S LCL, the durations of illness were recorded for 105 C LCL and 39 S LCL patients in adults (this information was missing for 1 S LCL patient) and for 69 C LCL (this information was missing for 3 C LCL patients) and 25 S LCL patients (this information was missing for 1 S LCL patient) in children.

^*^Statistical difference measured by Kruskal-Wallis

^#^ Statistical difference measured by Mann-Whitney
