# Supplementary material for: Demographic characteristics and clinical features of patients presenting with different forms of cutaneous leishmaniasis, in Lay Gayint, Northern Ethiopia
Source: PLoS Negl Trop Dis. 2024 Aug 15;18(8):e0012409. doi: 10.1371/journal.pntd.0012409 (PMC11349221; doi:10.1371/journal.pntd.0012409)
Supplement: S4 Table — The numbers of lesions in adult CL patients were recorded for 145 LCL, 49 MCL, 4 DCL, 1 recidivans CL and 8 multiple CL. For child CL patients, it was recorded for 98 LCL, 23 MCL, 1 DCL, 5 recidivans CL and 12 multiple CL. For the comparison between C LCL and S LCL, the numbers of lesions were recorded for 105 C LCL and 40 S LCL in adults and for 72 C LCL and 26 S LCL in children. CL = cutaneous leishmaniasis; C LCL = contained localised CL; S LCL: spreading localised CL; MCL = mucocutaneous CL; DCL = diffuse CL; RCL = recidivans CL.*Statistical difference measured by Kruskal-Wallis. # Statistical difference measured by Mann-Whitney. (DOCX) [file pntd.0012409.s004.docx]

**S4 Table: Number of lesions by CL type**

|  | **LCL** | **MCL** | **DCL** | **Recidivans** | **Multiple CL** | **p values*** |
| --- | --- | --- | --- | --- | --- | --- |
| **Adult** | 1 [1-2] | 1 [1-1] | 6 [6-6] | 1 [1-1] | 2 [2-3] | <0.0001 |
| **Children** | 1 [1-1] | 1 [1-3] | 6 [6-6] | 3 [3-3.5] | 3 [2-3.8] | 0.0002 |
|  | **C LCL** | **S LCL** | **p values^#^** |  |  |  |
| **Adult** | 1 [1-1] | 1 [2-3] | <0.0001 |  |  |  |
| **Children** | 1 [1-2] | 1 [2-2] | 0.1006 |  |  |  |

The numbers of lesions in adult CL patients were recorded for 145 LCL, 49 MCL, 4 DCL, 1 recidivans CL and 8 multiple CL. For child CL patients, it was recorded for 98 LCL, 23 MCL, 1 DCL, 5 recidivans CL and 12 multiple CL. For the comparison between C LCL and S LCL, the numbers of lesions were recorded for 105 C LCL and 40 S LCL in adults and for 72 C LCL and 26 S LCL in children.

^*^Statistical difference measured by Kruskal-Wallis

^#^ Statistical difference measured by Mann-Whitney
