# Supplementary material for: Demographic characteristics and clinical features of patients presenting with different forms of cutaneous leishmaniasis, in Lay Gayint, Northern Ethiopia
Source: PLoS Negl Trop Dis. 2024 Aug 15;18(8):e0012409. doi: 10.1371/journal.pntd.0012409 (PMC11349221; doi:10.1371/journal.pntd.0012409)
Supplement: S5 Table — Multiple locations: lesion on at least 2 of the following sites: face, ear, hand, neck, back, chest or leg. (DOCX) [file pntd.0012409.s005.docx]

**S5 Table: Location of lesions**

|  | **Face**  n (%) | **Ear**  n (%) | **Hand**  n (%) | **Thigh**  n (%) | **Shoulder**  n (%) | **Neck**  n (%) | **Multiple**  n (%) |
| --- | --- | --- | --- | --- | --- | --- | --- |
| **Adults** (n=207) | 178 (86) | 4 (1.9) | 6 (2.9) | 2 (1) | 0 | 2 (1) | 15 (7.2) |
| **Children** (n=139) | 128 (92) | 0 | 2 (1.4) | 0 | 1 (0.7) | 0 | 8 (5.9) |

Multiple locations: lesion on at least 2 of the following sites: face, ear, hand, neck, back, chest or leg
