# Supplementary material for: Demographic characteristics and clinical features of patients presenting with different forms of cutaneous leishmaniasis, in Lay Gayint, Northern Ethiopia
Source: PLoS Negl Trop Dis. 2024 Aug 15;18(8):e0012409. doi: 10.1371/journal.pntd.0012409 (PMC11349221; doi:10.1371/journal.pntd.0012409)
Supplement: S6 Table — Number of adult CL patients and their occupation and their education levels. *Students attending Orthodox Church schools. na = not applicable (DOCX) [file pntd.0012409.s006.docx]

**S6 Table: Occupation/education of adult CL patients**

| **Occupation**  **Education** | **Farmers**  **n (%)** | **Government employees**  **n (%)** | **Merchants**  **n (%)** | **Students**  **n (%)** |
| --- | --- | --- | --- | --- |
| **Total** | 149 (72) | 12 (5.8) | 4 (1.9) | 42 (20.3) |
| Illiterate | 87 (58.4) | na | na | na |
| Can write and read | 16 (10.7) | na | na | 2 (4.8)* |
| Primary school | 41 (27.5) | na | 3 (75) | 23 (54.8) |
| Secondary school | 2 (1.4) | 1 (8.3) | na | 8 (19) |
| College and above | 3 (2) | 11 (91.7) | 1 (25) | 9 (21.4) |

*Students attending Orthodox Church schools

na =not applicable
