# Supplementary material for: Demographic characteristics and clinical features of patients presenting with different forms of cutaneous leishmaniasis, in Lay Gayint, Northern Ethiopia
Source: PLoS Negl Trop Dis. 2024 Aug 15;18(8):e0012409. doi: 10.1371/journal.pntd.0012409 (PMC11349221; doi:10.1371/journal.pntd.0012409)
Supplement: S7 Table — Number of child adult patients and their education levels. (DOCX) [file pntd.0012409.s007.docx]

**S7 Table: Education of child CL patients**

|  | n (%) |
| --- | --- |
| Under school age (<7) | 24 (17.3) |
| Can write and read | 7 (5) |
| Primary/secondary school | 108 (77.7) |
