# Supplementary material for: Demographic characteristics and clinical features of patients presenting with different forms of cutaneous leishmaniasis, in Lay Gayint, Northern Ethiopia
Source: PLoS Negl Trop Dis. 2024 Aug 15;18(8):e0012409. doi: 10.1371/journal.pntd.0012409 (PMC11349221; doi:10.1371/journal.pntd.0012409)
Supplement: S8 Table — Number of patients who knew about CL, its transmission and treatment. *2 CL patients gave incorrect answers: transmission by bats or by direct contact. na = not applicable. (DOCX) [file pntd.0012409.s008.docx]

**S8 Table: Knowledge about CL, transmission and treatment**

|  | **Heard about CL** | |
| --- | --- | --- |
|  | Yes  n (%) | No  n (%) |
|  | 138 (66.7) | 69 (33.3) |
| Family | 72 (52.2) | na |
| Friend | 47 (34.1) | na |
| Health post | 5 (3.6) | na |
| Health centre | 3 (2.1) | na |
| School | 7 (5.1) | na |
| Hospital | 4 (2.9) | na |
|  | **Transmission** | |
|  | **No/incorrect**  n (%) | **Correct**  n (%) |
|  | 200* (96.6) | 7 (3.4) |
|  | **Treatment** | |
|  | Yes  n (%) | No  n (%) |
|  | 14 (6.8) | 193 (93.2) |
| Traditional healer | 7 (50) | na |
| Health post | 2 (14.2) | na |
| Health centre | 1 (7.2) | na |
| Hospital | 4 (28.6) | na |

*2 CL patients gave incorrect answers: transmission by bats or by direct contact. na=not applicable.
